# Exploring Potential Germline-Associated Roles of the TRIM-NHL Protein NHL-2 Through RNAi Screening

**DOI:** 10.1534/g3.117.300166

**Published:** 2017-08-17

**Authors:** Gregory M. Davis, Wai Y. Low, Joshua W. T. Anderson, Peter R. Boag

**Affiliations:** * Department of Biochemistry and Molecular Biology, Monash University, Clayton, Victoria 3800, Australia; † School of Applied and Biomedical Sciences, Federation University, Churchill, Victoria 3842, Australia; ‡ Development and Stem Cells Program, Monash Biomedicine Discovery Institute, Clayton, Victoria 3800, Australia

**Keywords:** NHL-2, TRIM-NHL, RNAi screen, germline, *Caenorhabditis elegans*, Mutant Screen Report

## Abstract

TRIM-NHL proteins are highly conserved regulators of developmental pathways in vertebrates and invertebrates. The TRIM-NHL family member NHL-2 in *Caenorhabditis elegans* functions as a miRNA cofactor to regulate developmental timing. Similar regulatory roles have been reported in other model systems, with the mammalian ortholog in mice, TRIM32, contributing to muscle and neuronal cell proliferation via miRNA activity. Given the interest associated with TRIM-NHL family proteins, we aimed to further investigate the role of NHL-2 in *C. elegans* development by using a synthetic RNAi screening approach. Using the ORFeome library, we knocked down 11,942 genes in wild-type animals and *nhl-2* null mutants. In total, we identified 42 genes that produced strong reproductive synthetic phenotypes when knocked down in *nhl-2* null mutants, with little or no change when knocked down in wild-type animals. These included genes associated with transcriptional processes, chromosomal integrity, and key cofactors of the germline small 22G RNA pathway.

TRIM-NHL proteins are highly conserved and are required for several biological processes including innate immunity, skeletal muscle plasticity and tumor suppression (Kudryashova *et al.* 2005; Liu *et al.* 2014; Nisole *et al.* 2005; Rajsbaum *et al.* 2008). In *Caenorhabditis* *elegans*, NHL-2 is one of five TRIM-NHL proteins and is orthologous to Brat and Mei-P26 in *Drosophila melanogaster* and TRIM32 in *Mus musculus* and *Homo sapiens* (Hammell *et al.* 2009; Loedige *et al.* 2014; Neumuller *et al.* 2008; Schwamborn *et al.* 2009). In flies, Mei-P26 is required for germline stem cell maintenance by regulating cellular proliferation and cell cycle exit via the microRNA (miRNA) pathway (Neumuller *et al.* 2008). In addition to this, Brat is required for tumor suppression in the larval brain and co-immunoprecipitates with AGO1, suggesting a miRNA-mediated role (Marchetti *et al.* 2014). In mammals, TRIM32 negatively regulates the tumor suppressor gene p53 via E3 ubiquitin ligase activity, suggesting that TRIM32 plays a role in tumorigenesis (Horn *et al.* 2004; Kano *et al.* 2008; Liu *et al.* 2014). Interestingly, TRIM32 positively regulates the miRNA pathway, and together with its E3 ligase activity is critical to regulating neuronal differentiation by regulating the transcription factor c-Myc (Schwamborn *et al.* 2009). These observations suggest diverse miRNA-associated roles for the TRIM-NHL subgroup of proteins in mammals and invertebrates.

In *C. elegans*, NHL-2 is required for embryonic polarity, asymmetric cell division, and sex determination (Hyenne *et al.* 2008; McJunkin and Ambros 2017). Moreover, NHL-2 functions as a miRNA cofactor to regulate developmental timing and cell fate progression (Hammell *et al.* 2009; Karp and Ambros 2012). In this role, NHL-2 associates with the DEAD-box RNA helicase CGH-1 to maintain miRNA function, and depletion of *cgh-1* in an *nhl-2* null background results in a let-7 defective phenotype (Hammell *et al.* 2009). In addition to this, Hammell *et al.* (2009) reported a temperature-sensitive reproductive defect in *nhl-2* null mutants. Therefore, we employed a synthetic RNAi screening approach to identify genes that result in strong reproductive phenotypes when knocked down in *nhl-2* mutants. In total, we identified 42 high-confidence candidate genes that produced defective reproductive phenotypes when knocked down in *nhl-2* null animals, but had little or no effect on wild-type animals. These candidate genes are associated with core biological functions such as cell cycle regulation, transcriptional processes, and DNA repair. We also identified several core cofactors of the germline-specific 22G RNA pathway. These genes represent potential candidate genes that associate with NHL-2 to maintain optimal reproduction, either through germline-specific small 22G RNAs, or through novel mechanisms.

## Materials and Methods

### Maintenance of C. elegans strains

*C. elegans* strains were obtained from the *C. elegans* Genetics Centre (CGC, Saint Paul, MN) and cultured under standard conditions (Brenner 1974). Strains used in this study were N2 (Bristol) as wild type and *nhl-2*(*ok818*), which was backcrossed eight times. Animals were grown while feeding on *Escherichia coli* OP50, seeded on NGM plates at 20°, unless otherwise stated.

### Synchronized populations of animals

To synchronize each strain, gravid adults were washed from NGM plates using M9 buffer (86 mM NaCl, 42 mM Na_2_HPO_4_, 22 mM KH_2_PO_4_, and 1 mM MgSO_4_), and animals were bleached by the addition of 1 ml of bleaching stock solution to each tube (1 ml of 10 M NaOH, 4 ml household bleach, and 9 ml of ddH_2_O). Tubes were then vortexed for 4 min and centrifuged at 1000 × *g*. Bleach was removed by four wash steps with sterile M9 buffer and eggs were allowed to hatch at 20° on a rotator.

### Liquid-based RNAi screen

The method used to screen for genes that produce synthetic phenotypes when knocked down in *nhl-2(ok818)* null mutants (referred to from here on as *nhl-2(0)*) was essentially as outlined by Lehner *et al.* (2006), with slight modifications as follows. RNAi was performed by feeding in duplicate 96 well plates for each strain using the ORFeome library (Reboul *et al.* 2003). Approximately 10 L1 animals of each strain in 10 μl M9 buffer with 0.01% Triton X-100 were added to each well using a multi-channel pipette. Plates were then placed in a shaking incubator at 150 rpm for 4 days at 20° and scored under a dissecting microscope for the following worm phenotypes: sterile (no progeny), embryonic lethality (unhatched embryos), low progeny (<20 progeny), very low progeny (<5 progeny), and egg-laying defective (eggs hatching inside animal). Phenotypes that were observed in the *nhl-2(ok818)* background, but not in wild-type animals, were considered as genes of interest. Positive hits were then rescreened using RNAi via plate feeding. Bacterial clones were grown in a solution of 2x TY media plus 100 μg/ml ampicillin overnight, then seeded onto NGM plates (3% bacto-agar, 86 mM NaCl, 42 mM Na_2_HPO_4_, 22 mM KH_2_PO_4_, and 1 mM MgSO_4_) containing 100 μg/ml ampicillin plus 4 mM IPTG. Approximately 10 synchronized L1 animals of each strain were then pipetted on to each plate in duplicates and grown at 20° for 4 days. Phenotypes were then scored under a dissecting microscope.

### Brood size assay

Brood size assays were performed for wild-type and *nhl-2(ok818)* strains with animals feeding on *E. coli* OP50. Synchronized populations of each strain were grown at 20° until the fourth larval stage (L4). Individual L4 animals were then placed on preseeded NGM plates, transferred to new plates every 12 hr, and scored for progeny after 48 hr. This process was repeated until each worm failed to lay new progeny. Total progeny included viable progeny and unhatched embryos.

### Germline dissection and immunostaining

Germline immunostaining was performed as described by Navarro *et al.* (2001). Anti-Phospho-Histone H3 antibody was applied at a 1:300 dilution (Abcam, Cambridge, England) and a secondary antibody and DAPI were applied at 1:1000 (Thermo Scientific, MA, USA). Slides were examined using an Olympus IX81microscope attached to an X-Cite series 120Q fluorescent light box.

### Bioinformatics

Potential homologs of hits from the RNAi screen were identified through BLASTP searches using *H. sapiens*, *D. melanogaster*, and *Saccharomyces cerevisiae* predicted proteomes available on the NCBI. Gene ontology (GO), was used to assign genes using GO Term Mapper (http://go.princeton.edu/cgi-bin/GOTermmapper) (Harris *et al.* 2004).

### Plasmid preparation and DNA Sequencing

To sequence genes from the secondary screen, bacteria expressing clones from the ORFeome library were grown overnight in 2x TY media plus 100 μg/ml ampicillin; plasmids were extracted by using the PureYield Miniprep System (Promega, Wisconsin, USA). Purified plasmids were then quantified by using a NanoDrop 2000 spectrophotometer (Thermo-Fischer Scientific, Massachusetts, USA) and sent to Macrogen for sequencing (http://dna.macrogen.com/eng/).

### Statistical analysis and software

Generation of graphs and statistical analysis was performed using the Prism 5 software package (GraphPad Software, California, USA). Images were processed using Adobe Photoshop (Adobe Systems, California, USA). Annotation of photos and generation of cartoon images used Adobe Illustrator (Adobe Systems).

### Data availability

The authors state that all data necessary for confirming the conclusions presented in the article are represented fully within the article.

## Results and Discussion

### Reproductive capacity of nhl-2(0) mutants

**Figure 1:**
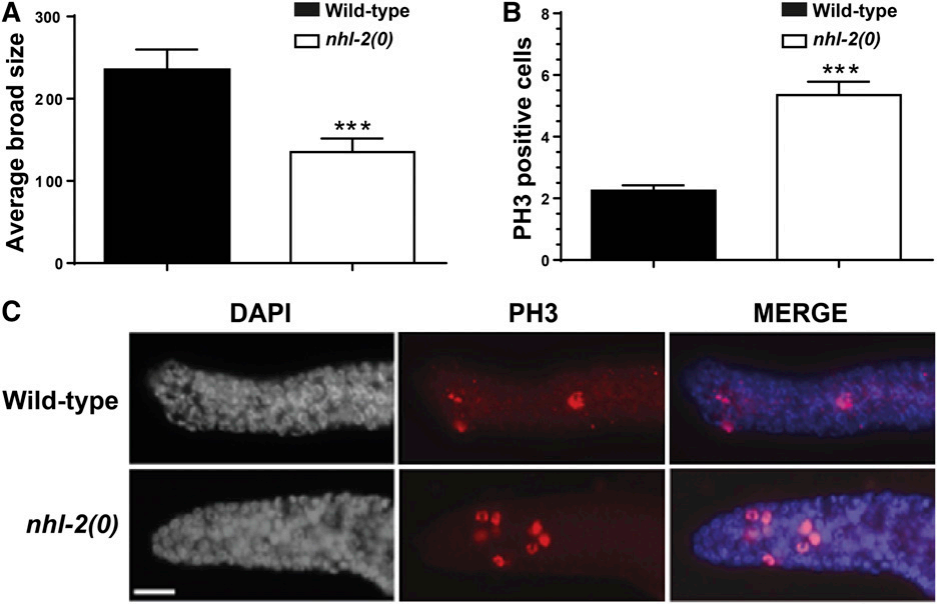
Brood size and elevated germ cell proliferation in *nhl-2(0)* mutants. (A) *nhl-2(0)* mutants display significantly reduced brood size when compared to wild-type animals at 20°. *** *P* <0.001, error bars represent SEM. *n* = 10. (B) Quantification of mitotic cells positive for PH3. *n* = 20. *** *P* <0.001, error bars represent SEM. (C) Represented images of germlines stained with PH3. Bar, 10 µm. *n* = 20.

The predicted *nhl-2(0)* mutant exhibits low penetrant heterochronic deficiencies and temperature-sensitive reproduction defects (Hammell *et al.* 2009). However, little evidence to date shows that NHL-2 plays a role in the *C. elegans* germline. To investigate the requirement of NHL-2 for germline function, a brood size assay was conducted in *nhl-2(0)* mutants and compared to wild-type animals. The *nhl-2(0)* mutants showed significantly reduced brood size when compared to wild-type animals at 20° (Figure 1A), suggesting that NHL-2 is required for normal reproductive function. To investigate potential *nhl-2(0)* germline defects, 1-day-old *nhl-2(0)* worms were dissected and the germlines immunostained with DAPI and an antibody specific for phosphorylation of histone 3 at serine 10 (PH3). This histone modification is required for correct chromosomal condensation during mitosis and is a suitable marker for mitotic proliferation (Hsu *et al.* 2000; Van Hooser *et al.* 1998). The *nhl-2(0)* germline morphology appeared grossly normal in appearance; however, analysis of the mitotic region of the germline revealed significantly higher numbers of cells that were positive for PH3 in *nhl-2(0)* mutants when compared to wild-type animals (Figure 1, B and C). This combination of reduced brood size and elevated mitotic proliferation prompted the use of an RNAi screen to identify potential cofactors that associate with NHL-2 to maintain normal germline function.

### Partial genome-wide RNAi screening in nhl-2(0) mutants

**Figure 2:**
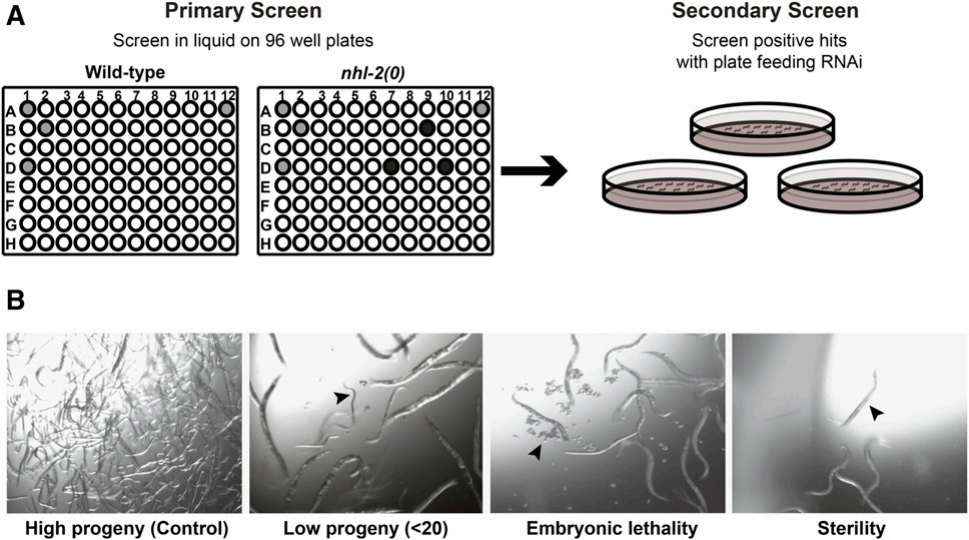
Genome-wide RNAi screening methodology. (A) Outline of primary liquid RNAi screen. Approximately 10 L1 wild-type or *nhl-2(0)* worms were deposited into each well of a 96-well plate that contained bacteria expressing dsRNA clones. Black wells represent phenotypes that are present in wild-type animals with no enhancement in *nhl-2(0)* mutants. Gray wells represent positive hits, where enhanced phenotypes were observed in *nhl-2(0)* mutants. Positive hits were then validated by plate feeding RNAi (secondary screen). (B) Representative DIC images of phenotypes as observed during the scoring process (arrows).

The RNAi screen used the ORFeome library that consists of 11,942 *C. elegans* open reading frames (∼55% of the *C. elegans* genome) (Reboul *et al.* 2003). Liquid feeding is the most efficient delivery method for large-scale RNAi screens (reviewed in Ahringer (2006)). Therefore, we optimized the appropriate volume of bacteria in 96-well plates to ensure that worms had sufficient food throughout the screen. Additionally, the appropriate orbital speed for shaking plates was assessed to guarantee maximum aeration for each well, but also to prevent cross-contamination between wells that contained different RNAi clones. Following this, our primary liquid feeding RNAi screen was performed at 20°, which resulted in 166 primary hits (Supplemental Material, Table S1 in File S1). These hits were defined as genes that gave synthetically defective reproductive phenotypes when knocked down in *nhl-2(0)* mutants when compared to knockdown of each gene in wild-type animals. These primary hits underwent a secondary screen where RNAi was delivered by plate feeding, resulting in 42 hits that displayed the same phenotypes as previously found in our primary screen (Figure 2). This final value of 42 genes resulted in a hit rate of 0.3% of the total ORFeome library. The most common phenotype identified was reduced progeny (24 hits), followed by embryonic lethality (14 hits) and sterility (four hits). Clones were sequenced for final confirmation prior to any further analysis. While some of the remaining 124 hits displayed abnormal germline phenotypes when knocked down in *nhl-2(0*) mutants, they produced phenotypes that were milder than those reported in our primary screen. This reduction in candidate genes from our primary to secondary screen is common in RNAi screens, and often a larger reduction in candidate genes has been observed in other screens (Maia *et al.* 2015; da Conceição Pereira *et al.* 2016; Saur *et al.* 2013). Nonetheless, the final 42 candidate genes identified through both liquid and plate feeding RNAi approaches produced consistent phenotypes. The robustness of our screen was also supported by the independent identification of *mpk-1*, which is duplicated in the ORFeome library and consistently resulted in low progeny when knocked down in *nhl-2(0)* mutants, but not in wild-type animals. We cannot rule out the possibility that some of these hits may not be specific to *nhl-2*, and represent genes sensitive to germlines with disrupted homeostasis. However, our final screen results represent candidate genes that are worthy of further investigation to elucidate novel roles for NHL-2.

### Characterization of genes

Our primary screen in liquid culture identified several genes associated with pathways that regulate mitotic proliferation. These included the gene encoding the PUF protein, *fbf-1*, and the Notch signaling components *lag-1*, *nos-3*, and *sel-12* (Hansen *et al.* 2004; Henderson *et al.* 1994; Kadyk and Kimble 1998; Lambie and Kimble 1991; Levitan and Greenwald 1995; Wen *et al.* 2000; Zhang *et al.* 1997). However, these genes were excluded from our high-confidence list as they were only mildly enhanced in *nhl-2(0)* mutants compared to knock down in wild-type animals. In addition to this, we assessed whether the enhanced mitotic proliferation observed in *nhl-2(0)* mutants was enhanced in the synthetic phenotypes observed in our high-confidence hit list. Unfortunately, no trend was observed when each gene was knocked down in *nhl2(0)* mutants and immunostained for PH3 (Figure S1 in File S1). This suggests that the genes associated with our final results are not primarily associated with mitotic proliferation.

To determine any trends associated with the 42 high-confidence screen hits, we further classified these based on their homology, their germline enrichment, and their NCBI Clusters of Orthologous Groups (COGs) description (WormBase, WS204). GO terms for molecular function, cellular compartment, and biological process were analyzed by GO Term Mapper (Harris *et al.* 2004); however, this analysis did not show any clear trend, possibly due to the small number of high-confidence genes (Table S2, Table S3, and Table S4 in File S1). Therefore, genes were grouped based on their reported function, as well as whether each gene was enriched in the germline (WormBase, WS204). This information was then used to group the final secondary screen results based on criteria for each gene (Table 1). However, RNAi by feeding in our laboratory conditions did produce negative results for genes with previously reported lethal phenotypes. An example of this is *cgh-1*, which results in sterility when knocked down in wild-type animals (Navarro *et al.* 2001), yet unexpectedly did not register as a positive hit in our screen. This suggests that high-throughput RNAi screening does have limitations. Nonetheless, our final high-confidence gene list showed consistent phenotypes using both liquid and plate feeding RNAi approaches.

**Table 1:** Genes from secondary screen listed per reported function

Gene (Phenotype)	Description (NCBI COGs)	Enriched	Homology		
			H	D	S
Transcriptional/nuclear organization hits					
* mdt-8* (Lp)	Mediator of RNA polymerase II transcription subunit 8	Sp			
* mdt-10* (Lp)	Mediator of RNA polymerase II transcription subunit 10	Sp & Oo			
* mdt-27* (Lp)	Mediator of RNA polymerase II transcription subunit 27	Sp & Oo			
* mrg-1* (Ste)	Chromodomain-containing protein	Sp			
* ntl-3* (Ste)	CCR4-NOT transcriptional regulation complex				
C36E8.1 (Ste)	RNA polymerase I transcription factor	Sp & Oo			
* spe-44* (Lp)	Nuclear DEAF-1 related transcriptional regulator	Sp & Oo			
* pqn-85* (Lp)	Sister chromatid cohesion protein	Sp & Oo			
F58G1.2 (Lp)	Zn-finger protein	Sp			
* fem-1* (Lp)	Ankyrin repeat protein	Sp & Oo			
Y54G2A.26 (Lp)	Unknown (transcriptional corepressor in *S. pombe*).				
* sas-1* (Emb)	Spindle assembly protein	Sp			
Cell cycle regulation					
* cya-1* (Lp)	G2/mitotic-specific cyclin A	Sp & Oo			
* orc-5* (Emb)	Origin recognition complex	Sp & Oo			
F35G12.11 (Lp)	Enhancer of rudimentary homolog	Sp			
Motor related proteins					
* arp-11*(Emb)	Actin related protein	Sp & Oo			
* dli-1* (Emb)	Dynein light intermediate chain	Sp & Oo			
* dnc-1**^a^* (Lp)	Microtubule-associated protein	Sp & Oo			
* mop-25.2**^a^* (Lp)	Conserved mouse embryo scaffolding protein	Sp & Oo			
F28D1.2 (Lp)	Myosin 1 homolog	Sp & Oo			
Microtubule-related proteins					
* dnc-1**^a^* (Lp)	Microtubule-associated protein	Sp & Oo			
* pfd-1* (Lp)	Molecular chaperone Prefoldin subunit 1				
* pfd-2* (Emb)	Molecular chaperone Prefoldin subunit 2	Sp & Oo			
Chromosomal integrity and DNA repair					
* smz-2* (Emb)	Sperm meiosis PDZ domain containing protein	Sp			
* drh-3* (Emb)	DEAD-box RNA helicase	Sp & Oo			
* cde-1* (Emb)	Germline-specific nucleotidyltransferase protein				
* ekl-1* *^a^*(Emb)	Tudor domain protein				
* spdl-1* (Lp)	Coiled-coil protein	Sp & Oo			
* mop-25.2**^a^* (Lp)	Conserved mouse embryo scaffolding protein	Sp & Oo			
* rad-51* (Emb)	DNA repair protein	Sp & Oo			
* him-14*(Emb)	MutS family of DNA mismatch repair protein	Sp & Oo			
* dsb-1* (Lp)	Double-strand break factor	Sp & Oo			
Mitochondrial related proteins					
* lpl-1* (Ste)	Lipoate ligase homolog	Sp			
* mtch-1* (Lp)	Mitochondrial carrier homolog 1	Sp&Oo			
Kinase proteins					
C56A3.8 (Lp)	Phosphatidylinositol 4-kinase type 2-alpha homolog	Sp & Oo			
* mpk-1* (Lp)	Mitogen-activated protein kinase	Sp			
Other biological functions					
C06A8.6 (Lp)	Ortholog of human 1RRC9	Sp & Oo			
C34D4.4 (Lp)	Ortholog of human TVP23A	Sp & Oo			
R05H5.3 (Emb)	Ortholog of human NXNl2	Sp & Oo			
* unc-11* (Lp)	Clathrin assembly protein	Sp			
T12A2.7 (Lp)	Ortholog of human BCAS2	Oo			
Proteins with unknown function					
C46A5.5 (Lp)	Unknown	Sp			

Phenotypes in *nhl-2(ok818)* mutants: Lp, low progeny; Ste, sterile; Emb, embryonic lethal. Gray = BLAST e value <0.1 and >30% query coverage. Black = BLAST e value <0.1 and >20% query coverage. White = BLAST e value >0.1. Sp = sperm enriched, Oo = oocyte enriched. Homology: H = *H. sapiens*, D = *D. melanogaster*, S = *S. cerevisiae*.

a Genes with >1 reported function.

Interestingly, 12 hits were associated with transcriptional/nuclear organization processes, as well as nine genes required for chromosomal integrity or DNA repair. We identified hits associated with components of the DNA damage repair pathway, such as *rad-51* and *dsb-1* (Stamper *et al.* 2013; Takanami *et al.* 2003), as well as the DNA mismatch repair gene *him-14* (Zalevsky *et al.* 1999). Another subset of genes with reported chromosomal association were components of the small 22G RNA pathway. These included the DEAD-box RNA helicase *drh-3*, the tudor domain protein *ekl-1*, and the nucleotidyltransferase *cde-1* (Gu *et al.* 2009; Nakamura *et al.* 2007; van Wolfswinkel *et al.* 2009). Interestingly, many of the genes associated with chromosomal integrity, DNA repair, and the 22G RNA pathway displayed enhanced synthetic embryonic lethality when knocked down in *nhl-2(0)* mutants. Given the association of the 22G RNA pathway with centromere formation in early embryogenesis (Claycomb *et al.* 2009), and the presence of DNA damage repair genes in our screen, it is possible that NHL-2 may have a role associated with embryonic chromosome integrity.

As expected, the majority of genes from our screen were strongly enriched within the germline (Baugh *et al.* 2003; Reinke *et al.* 2004). This consisted of 26 genes enriched in both oocytes and sperm, and 10 genes enriched in sperm only. This resulted in a total of 38 genes that were germline enriched and produced synthetically enhanced germline-deficient phenotypes when knocked down in *nhl-2(0)* mutants. The high rate of genes enriched in sperm and oocytes suggests that these synthetic phenotypes are the result of deficiencies associated with central germline processes.

The majority of NHL-2 screen hits are highly conserved. BLAST results grouped genes based on homology against *H. sapiens*, *D. melanogaster*, and *S. cerevisiae*. Our criteria for homology was as follows: high homology, where genes scored e-values <0.1% and query coverage of >30%; and moderate homology, where genes scored e-values <0.1% with query coverage >20%. Based on these criteria, the homology of our final hits was considerably high, with only four genes being nematode-specific genes. This is highly relevant, as it is anticipated that this investigation will provide additional information that is relevant for orthologs of NHL-2 in higher organisms. In all, this synthetic RNAi screen identified genes associated with candidate pathways that may require NHL-2, which are highly enriched in the germline. Given the diverse nature of TRIM-NHL proteins, including those with multiple functions, this screen may lead to the identification of novel roles for NHL-2.

## Supplementary Material

Supplemental material is available online at www.g3journal.org/lookup/suppl/doi:10.1534/g3.117.300166/-/DC1.
