# Supplementary material for: Exploring Potential Germline-Associated Roles of the TRIM-NHL Protein NHL-2 Through RNAi Screening
Source: G3 (Bethesda). 2017 Aug 17;7(10):3251–6. doi: 10.1534/g3.117.300166 (PMC5633376; doi:10.1534/g3.117.300166)
Supplement: Supplementary file 1 [file 3251FileS1.pdf]

SUPPLEMENTARY MATERIAL:

Exploring potential germline associated roles of the TRIM-NHL protein NHL-2 through RNAi screening

Gregory M. Davis[1, 3], Wai Y. Low[1, #], Joshua W.T. Anderson[1, 2], and Peter R. Boag[1, 2,*]

[1] Department of Biochemistry and Molecular Biology, Monash University, Wellington Road, Clayton, VIC 3800, Australia; [2] Development and Stem Cells Program, Monash Biomedicine Discovery Institute, Wellington Road, Clayton, VIC 3800, Australia; [3] School of Applied and Biomedical Sciences, Federation University, Northways Road, Churchill VIC 3842, Australia.

Keywords: NHL-2, TRIM-NHL, RNAi screen, germline, *Caenorhabditis elegans*

[*] Corresponding author: email peter.boag@monash.edu

[#] Current address: The Davies Research Centre, School of Animal and Veterinary Sciences, University of Adelaide, Roseworthy, SA 5371, Australia

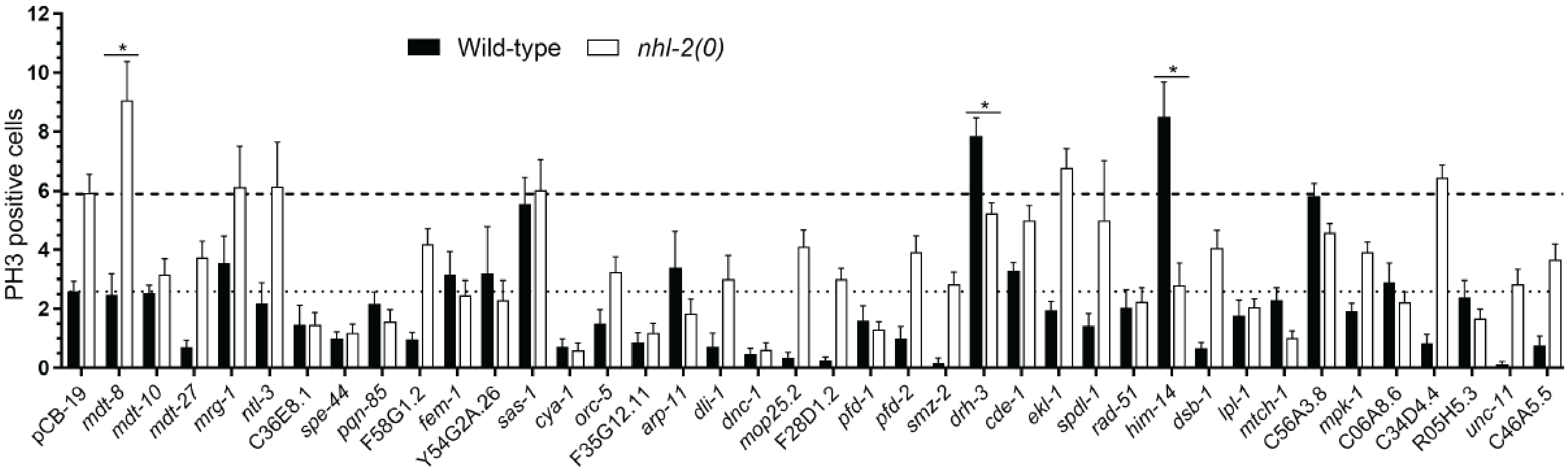

Figure S1: Germ cell proliferation phenotypes of RNAi screen hits in *nhl-2(0)* mutants

Knockdown of high confidence genes as listed in Table 1 immunostained and scored for PH3 positive cells. pCB-19 represents mock-treated RNAi as a positive control. Lower dotted line represents PH3 values in wild-type animals and top dashed line represents PH3 values in mock-treated *nhl-2(0)* mutants. Significance was assigned only when knockdown of a gene was significantly different that observed in *nhl-2(0)* alone. * = P values <0.05, error bars represent standard error of mean.

Table S1: Primary RNAi screen results (Hp = high progeny, S = sterile, El = embryonic lethality, Lp = low progeny, La = larval arrest)

Gene ID	wild-type/*nhl-2(0)* phenotypes
K06H7.6	Hp/S
ZK287.5	Hp/S
B0464.5c	Mp/S
C18D4.6a	Hp/El
C32E8.10d	Hp/S
C32E8.10e	Hp/S
D2005.5	Hp/El
F32H2.1b	Mp/S
F36H1.4c	Hp/La
F43C1.2b	Hp/S
F44A6.2b	Hp/El
F56F3.2	Hp/El
F58D5.4b	Hp/S
M88.6b	Mp/La
R06F6.5b	Hp/El
W07B3.2d	Mp/La
W09C5.6b	Mp/S
Y17G7B.15b	Hp/S
Y47D3A.6a	Mp/La
ZC410.7a	Hp/El
ZK520.4c	Mp/S
B0024.9	Hp/El
B0205.6	Hp/S
B0250.2	Hp/Lp
B0280.10	Hp/Lp
B0412.1	Hp/Lp
B0432.3	Hp/S
C01G10.8	Hp/S
C01H6.5b	Hp/El
C05B5.3	Hp/S
C06A8.5	Hp/Lp
C06A8.6	Hp/Lp
C07H6.8	Hp/El
C08F8.1	Hp/El
C09G4.3	Hp/El
C14B1.7	Hp/El
C25G4.4	Hp/Lp
C27C12.7	Hp/S
C30G12.7	Hp/S
C32A3.1	Hp/S
C32E8.10	Hp/Lp
C33H5.4	Hp/El
C34B2.8	Hp/Lp

C34C12.8	Mp/S
C34D4.4	Hp/Lp
C35E7.9	Hp/Lp
C36B1.1c	Hp/S
C36E8.1	Hp/La
C37A5.9	Hp/Lp
C39E9.14	Hp/El
C46A5.5	Hp/Lp
C56A3.8	Hp/Lp
D1081.5	Hp/Lp
D2013.8	Hp/El
E01A2.6	Hp/Lp
F08F1.9	Hp/La
F08G5.1	Hp/Lp
F09G8.2	Hp/Lp
F11G11.2	Hp/El
F12F6.5	Hp/S
F16C3.1	Hp/Lp
F18A1.4	Hp/La
F20H11.3	Mp/S
F21H12.5	Mp/El
F22B8.7	Hp/El
F22D6.2	Hp/Lp
F22D6.6	Hp/El
F22F4.2	Mp/El
F23H11.4	Hp/El
F23H11.7	Hp/Lp
F23H12.2	Hp/S
F26F4.11	Mp/S
F28D1.2	Hp/La
F29B9.6	Mp/S
F29D10.5	Hp/Lp
F31C3.5	Hp/El
F32H2.1	Hp/S
F33E2.5	Hp/Lp
F33H1.3	Hp/El
F35D6.1a	Hp/La
F35H12.3	Hp/Lp
F36A2.7	Mp/S
F37C12.1	Hp/Lp
F43C1.2	Hp/Lp
F43E2.7	Hp/Lp
F46A9.4	Hp/El
F52F12.4	Hp/El
F54C1.9	Hp/Lp
F54D7.2	Hp/Lp
F57B10.8	Hp/Lp

F58A4.9	Mp/S
F58D5.4b	Hp/El
F58G1.2	Hp/Lp
F59A3.3	Hp/S
H20J04.5	Hp/El
H27M09.1	Hp/El
K01A2.5	Hp/S
K08B4.1	Mp/lp
K08E3.5	Hp/Lp
K08E7.3	Hp/El
K10D2.3	Mp/El
K11B4.1	Hp/S
K11H3.3	Hp/Lp
M7.5	Hp/S
R05D7.4	Hp/Lp
R05H5.3	Mp/El
R06C1.3	Hp/El
R09B3.4	Hp/El
R09B5.8	Hp/El
R10H10.2	Hp/S
R11A5.4	Hp/Lp
R12B2.5	Mp/Lp
T01G9.6	Hp/S
T02E1.6	Hp/Lp
T04A8.2	Hp/Lp
T05E11.3	Hp/La
T05F1.4	Hp/Lp
T09A5.5	Hp/S
T09A5.6	Hp/Lp
T09A5.9	Hp/S
T15B12.1	Hp/S
T16H12.3	Hp/S
T17H7.4d	Hp/La
T18H9.6	Hp/Lp
T20B12.2	Hp/S
T20H9.2	Hp/Lp
T21G5.4	Hp/El
T23F11.3	Hp/Lp
T26A5.7	Hp/La
W01G7.3	Mp/S
W02D3.9	Hp/El
W06E11.2	Hp/S
Y111B2A.24	Hp/El
Y37D8A.9	Hp/S
Y39A3A.1	Hp/Lp
Y39E4B.10	Hp/S
Y39E4B.9	Hp/Lp

Y43C5A.6	Hp/El
Y43H11AL.3	Hp/Lp
Y4C6B.3	Hp/Lp
Y53C12A.4	Hp/Lp
Y53C12B.3b	Mp/lp
Y54G2A.26	Hp/Lp
Y56A3A.1	Hp/S
Y56A3A.20	Hp/S
Y62F5A.1	Hp/Lp
Y6B3B.9	Hp/S
Y70G10A.2	Hp/Lp
Y74C10AR.1	Mp/La
Y75B8A.35	Mp/S
Y79H2A.6	Hp/La
Y95B8A.11	Hp/Lp
ZC168.3	Hp/El
ZC477.9c	El/S
ZK1010.3	Hp/La
ZK1058.3	Hp/Lp
ZK1098.2a	Hp/S
ZK1127.11	Hp/S
ZK1127.5	Hp/La
ZK1307.5	Mp/El
ZK507.6	Hp/Lp
ZK512.2	Mp/S
ZK512.6	Hp/El
ZK525.2	Hp/Lp
ZK593.5	Hp/Lp
R17.1	Hp/S

Table S2: Gene ontology (GO terms) for genes associated with biological processes

GO Terms (biological process)	Genes
anatomical structure development (GO:0048856)	33
embryo development (GO:0009790)	31
reproduction (GO:0000003)	27
cellular nitrogen compound metabolic process (GO:0034641)	11
cell cycle (GO:0007049)	11
locomotion (GO:0040011)	9
biosynthetic process (GO:0009058)	8
cell differentiation (GO:0030154)	8
cell death (GO:0008219)	8
chromosome segregation (GO:0007059)	7
transport (GO:0006810)	6
growth (GO:0040007)	6
cellular component assembly (GO:0022607)	5
response to stress (GO:0006950)	5
vesicle-mediated transport (GO:0016192)	5
mitotic nuclear division (GO:0007067)	4
macromolecular complex assembly (GO:0065003)	4
chromosome organization (GO:0051276)	4
catabolic process (GO:0009056)	4
DNA metabolic process (GO:0006259)	3
signal transduction (GO:0007165)	3
cytoskeleton organization (GO:0007010)	3
protein complex assembly (GO:0006461)	3
aging (GO:0007568)	3
cellular protein modification process (GO:0006464)	3
cytoskeleton-dependent intracellular transport (GO:0030705)	2
protein folding (GO:0006457)	2
cell morphogenesis (GO:0000902)	2
cell division (GO:0051301)	2
developmental maturation (GO:0021700)	1
nucleobase-containing compound catabolic process (GO:0034655)	1
translation (GO:0006412)	1
anatomical structure formation involved in morphogenesis (GO:0048646)	1
small molecule metabolic process (GO:0044281)	1
cell proliferation (GO:0008283)	1
cell motility (GO:0048870)	1
membrane organization (GO:0061024)	1

Table S3: Gene ontology (GO terms) for genes associated with molecular function

GO Terms (molecular function)	Genes
DNA binding (GO:0003677)	3
transcription factor activity, protein binding (GO:0000988)	3
ion binding (GO:0043167)	2
ATPase activity (GO:0016887)	2
kinase activity (GO:0016301)	2
unfolded protein binding (GO:0051082)	2
enzyme binding (GO:0019899)	2
RNA binding (GO:0003723)	1
helicase activity (GO:0004386)	1
ligase activity (GO:0016874)	1
signal transducer activity (GO:0004871)	1
cytoskeletal protein binding (GO:0008092)	1
nucleotidyltransferase activity (GO:0016779)	1
nucleic acid binding transcription factor activity (GO:0001071)	1
nuclease activity (GO:0004518)	1
lipid binding (GO:0008289)	1

Table S4: Gene ontology (GO terms) with genes associated with cellular compartment

GO Terms (cellular compartment)	Genes
cell (GO:0005623)	28
intracellular (GO:0005622)	28
organelle (GO:0043226)	26
nucleus (GO:0005634)	15
cytoplasm (GO:0005737)	14
protein complex (GO:0043234)	13
chromosome (GO:0005694)	12
cytoskeleton (GO:0005856)	6
nuclear chromosome (GO:0000228)	6
nucleoplasm (GO:0005654)	4
microtubule organizing center (GO:0005815)	2
Golgi apparatus (GO:0005794)	2
endoplasmic reticulum (GO:0005783)	1
cytoplasmic vesicle (GO:0031410)	1
cytosol (GO:0005829)	1
mitochondrion (GO:0005739)	1
plasma membrane (GO:0005886)	1
